# The Balance of Th17 versus Treg Cells in Autoimmunity

**DOI:** 10.3390/ijms19030730

**Published:** 2018-03-03

**Authors:** Gap Ryol Lee

**Affiliations:** Department of Life Science, Sogang University, 35 Baekbeom-ro, Mapo-gu, Seoul 04107, Korea; grlee@sogang.ac.kr; Tel.: +82-2-705-8458; Fax: +82-2-704-3601

**Keywords:** Th17, Treg, balance, autoimmunity, RORγt, Foxp3

## Abstract

T helper type 17 (Th17) cells and pTreg cells, which share a common precursor cell (the naïve CD4 T cell), require a common tumor growth factor (TGF)-β signal for initial differentiation. However, terminally differentiated cells fulfill opposite functions: Th17 cells cause autoimmunity and inflammation, whereas Treg cells inhibit these phenomena and maintain immune homeostasis. Thus, unraveling the mechanisms that affect the Th17/Treg cell balance is critical if we are to better understand autoimmunity and tolerance. Recent studies have identified many factors that influence this balance; these factors range from signaling pathways triggered by T cell receptors, costimulatory receptors, and cytokines, to various metabolic pathways and the intestinal microbiota. This review article summarizes recent advances in our understanding of the Th17/Treg balance and its implications with respect to autoimmune disease.

## 1. Introduction

The immune system provides essential protection against pathogenic microorganisms; however, it may also attack self tissue. CD4 T cells coordinate diverse immune responses to deal with various disease-causing pathogens. Naïve CD4 T cells are activated upon binding of the T cell receptor (TCR) and a costimulatory receptor (CD28) to the peptide–major histocompatibility complex (MHC) complex and a costimulatory molecule (B7.1 or B7.2), respectively; the latter are expressed/presented by antigen-presenting cells (APCs). Activated CD4 T cells differentiate into several subsets of effector cells that have different functions. These cell types include T helper type I (Th1), Th2, Th17, T follicular helper (Tfh), and regulatory T (Treg) cells [1]. The final fate is primarily determined by the external milieu (e.g., cytokines) present during activation. Th1 cells stimulate classical macrophages and mediate immune responses to intracellular pathogens. Th2 cells stimulate mast cells, eosinophils, and basophils, and mediate immune responses to helminths. Th17 cells stimulate many cell types to recruit neutrophils to sites of infection, and mediate immune responses against extracellular bacteria and fungi. Tfh cells stimulate B cell maturation at germinal centers. Treg cells inhibit immune responses to maintain immune homeostasis. Among the subsets, Th1 and Th17 cells can cause, but Treg cells suppress, autoimmune disease. Here, I will focus the role of Th17 cells and Treg cells in autoimmunity.

## 2. The Th17/Treg Balance in Autoimmunity

Th17 cells and Treg cells share a common signaling pathway mediated by TGF-β. However, proinflammatory signals present during cell activation regulate the fate of these cells reciprocally [1,2]. For example, in the presence of interleukin (IL)-6 or IL-21 (together with TGF-β), naïve CD4^+^ T cells differentiate into Th17 cells; however, in the absence of proinflammatory cytokines, TGF-β drives differentiation into Treg cells [3,4,5]. Th17 cells produce IL-17, IL-22, and IL-23, recruit neutrophils, and promote inflammation at the infection site. By contrast, Treg cells produce anti-inflammatory cytokines IL-10 and TGF-β, suppress activity of a variety of immune cells, and thereby inhibit immune responses. Thus, these two cell types play opposite roles during inflammatory and immune responses [6]. Th17 cells are a major player in autoimmune diseases, such as psoriasis, inflammatory bowel disease (IBD), rheumatoid arthritis (RA), and multiple sclerosis (MS). STAT3 is activated upon stimulation by TCR/costimulatory signals, together with TGF-β and IL-6 [3,4,5]; STAT3 then induces expression of transcription factor RAR-related orphan receptor (ROR)γt, which derives cells toward the Th17 subset [7].

By contrast, Treg cells inhibit autoimmune responses. Treg cells arise either during thymic development, or in the periphery, via activation of naïve CD4 T cells. Thymus-derived Treg cells are called tTreg cells, whereas periphery-derived Treg cells are called pTreg cells. tTreg cells are generated by receiving relatively strong TCR stimulation by self-antigen-MHC complexes on thymic APCs, which leads to Foxp3 expression. On the other hand, pTreg cells are generated from naive CD4 T cells by receiving antigen stimulation under the influence of TGF-β and IL-2 in the periphery. pTreg cells are prevalent in certain organs, such as the gut and maternal placenta. Thus, pTreg cells are considered to play an important role in maintaining tolerance against foods, commensal bacterial, and the fetus during pregnancy. Stimulation of naïve CD4 T cells with TGF-β induces SMAD2 and SMAD3, which in turn activate transcription factor Foxp3; this drives cells toward the pTreg lineage [8]. In addition, IL-2 induces STAT5, which activates Foxp3. Foxp3^+^ Treg cells can also be induced by TGF-β and IL-2 in vitro; these cells are called iTreg cells. Other types of cells that have suppressive functions are Th3, T regulatory type 1 (Tr1), and glucocorticoid-induced TNFR-related protein (GITR) single-positive (GITRsp) cells [9,10,11]. Th3 cells produce TGF-β, and are Foxp3^+^ [9]; Tr1 cells produce IL-10, and are Foxp3^−^ [10]; and GITRsp cells express GITR but low CD25, and produce high amounts of IL-10 and TGF-β [11]. I will focus on the CD4^+^Foxp3^+^ Treg cells in this review.

Th17 cells cause, whereas Treg cells inhibit, autoimmunity. Thus, the reciprocal generation of Th17 and Treg cells is critically important. Many factors that influence the generation and maintenance of these cells are also important for appropriate regulation of the Th17/Treg balance; these include TCR signals, costimulatory signals, cytokine signals, Foxp3 stability, metabolic processes, and the microbiota. Below, I explain what we know about these factors, their role(s) in regulating the Th17/Treg balance, and the implications for development of autoimmunity.

## 3. TCR Signaling

**Figure 1:**
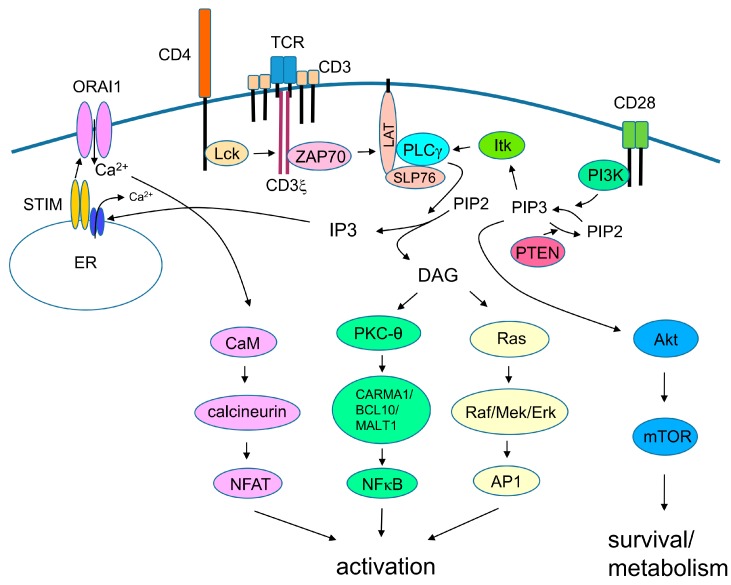
Signaling pathways of T cell receptor (TCR) and costimulatory signal. Simplified view of signaling pathways through TCR and costimulatory signal. TCR binds to peptide–MHC and CD28 binds to B7.1 or B7.2 on the surface of APC. Signaling pathways ultimately induce activation and survival of T cells.

Signaling via the TCR is required for activation of naïve T cells (Figure 1). When the TCR, together with co-receptors CD4 or CD8, binds to the peptide–MHC complex on the surface of APCs, the immunoreceptor tyrosine-based activation motifs (ITAMs) in the cytoplasmic tails of the CD3 chains are located in close proximity to protein kinase Lck, which is associated with the cytoplasmic tail of the co-receptor; this results in phosphorylation of the ITAMs. A phosphorylated ITAM recruits the protein kinase Zap70, which then phosphorylates adaptor molecules SLP-76 and LAT. TCR/costimulatory signals also activate PI3K, which generates phosphatidylinositol (3,4,5)-trisphosphate (PIP_3_) from PI(4,5)P_2_. PIP_3_ recruits PH domain-containing proteins such as Akt, IL-2 inducible T-cell kinase (Itk), phospholipase C (PLC)-γ, guanine nucleotide exchange factor Vav, and adhesion and degranulation promoting adaptor protein (ADAP) on the cell membrane. PLC-γ binds to the SLP-76/Gads/linker for activated T cells (LAT) complex, and is phosphorylated and activated by Itk. Activated PLC-γ cleaves the membrane lipid PIP_2_ to yield DAG and IP_3_. IP_3_ binds to a calcium channel on the endoplasmic reticulum (ER) and promotes release of Ca^++^ from the ER reservoir. Ca^2+^ release stimulates oligomerization of stromal interaction molecule (STIM) on the ER membrane, and subsequently activates plasma membrane Ca^2+^ channel ORAI1, which eventually increases the Ca^2+^ concentration in the cytoplasm. Ca^2+^ acts on the calmodulin/calcineurin pathway, which activates the transcription factor NFAT. DAG activates two signaling pathways: the Ras/Raf/MAPK pathway (which eventually induces transcription factor AP-1) and PKC-θ, to stimulate formation of the CARMA1/BCL10/MALT1 complex, which then recruits and activates the TRAF-6/TAK1/IKK pathway and, eventually, activates transcription factor NF-κB.

Accumulated evidence shows that the strength of the TCR signal affects tTreg cell development in the thymus [12]. Attenuated TCR signals, caused by deficiency or mutation of TCR signaling components, such as Zap70 [13], LAT [14,15], PLCγ1 [16], STIM [17], DAG [18,19], Raf [20,21], or NF-κB [22,23,24,25], results in defective tTreg cell development. However, in contrast to these results, attenuated TCR signal can promote tTreg cell generation. A phosphorylation-deficient CD3ζ mutant, which shows attenuated TCR signaling, promotes tTreg cell generation [26]. Indeed, Akt activity is increased in CD3ζ mutant T cells [26]. Akt activates mechanistic target of rapamycin (mTOR), which in turn inhibits Treg cell generation. Thus, development of tTreg cells may require a TCR signal of a certain strength or a particular method of TCR ligation [12].

Whether TCR signal strength also regulates pTreg and iTreg cell differentiation is less well understood. Although differentiation of iTreg cells requires IKK and Ca^2+^ signals [27,28,29], it also requires a weak signal from the TCR [30]. Attenuated TCR signaling in Itk^−/−^ cells activates phosphatase and tensin homolog (PTEN) and inhibits the Akt/mTOR pathway, resulting in preferential differentiation into Treg cells rather than Th17 cells [30]. Although activation of PTEN drives Treg cell differentiation, diminished expression of PTEN is also detrimental for Th17 cell differentiation because it increases IL-2 production [31]; this suggests that extreme PTEN levels (low or high) disrupt the Th17/Treg balance. Thus, similar to mTOR, it seems that a certain PTEN level is required to maintain an appropriate Th17/Treg cell balance. It is not clear why tTreg and iTreg cells need different TCR signal strengths or a particular method of TCR ligation for development/differentiation; nonetheless, what we do know is that the TCR signal is important for Treg cell development and for reciprocal regulation of Treg versus Th17 differentiation.

The TCR signal is also important for suppressive function of Treg cells. Deficiency or mutation of TCR signaling components Zap70, LAT, STIM, and NF-κB leads to reduced suppressor activity [15,17,32,33]. Besides activating NF-κB and NFAT, the TCR signal activates Akt, which in turn suppresses Foxo1. Foxo1 is required to maintain Treg suppressive activity as it suppresses IFN-γ and induces transcription factor Klf2 and chemokine receptor CCR7 [34].

Proteins that modify or fine-tune the TCR signaling pathway also influence the Th17/Treg balance. SHARPIN, a ubiquitin-binding and ubiquitin-like-domain-containing protein, promotes Treg cell differentiation, but prevents conventional T cell differentiation by inhibiting the interaction between TCRζ and Zap70 [35]. Deletion or pharmacological inhibition of protein kinase casein kinase 2 (CK2) blocks Th17 differentiation and induction of Treg cells [36,37,38]. Deleting or inhibiting CK2 dampens the STAT3 and mTOR signaling pathways [36,37,38]. Protein phosphatase 2A (PP2A) regulates mTORC1 activity. Treg cell-specific deletion of PP2A causes severe multi-organ autoimmune disease, suggesting that PP2A is required for Treg cell function [39].

## 4. Costimulatory Signals

Activation and differentiation of naïve T cells requires costimulatory signals from APCs. Costimulatory molecules B7.1 (CD80) and B7.2 (CD86) on the APC surface bind to CD28 on naïve T cells. Costimulatory signals are thought to amplify the TCR signal. The cytoplasmic tail of CD28 contains docking sites for signaling molecules [40]. The membrane proximal YMNM motif binds to PI3K, and the distal PYAP motif binds to Grb2 and Lck [40].

Costimulatory signals are required for both tTreg and pTreg cell generation; indeed, mice deficient in CD28 signaling harbor reduced numbers of both Treg types, leading to autoimmune diseases [41]. CD28 signaling is essential for homeostasis and function of peripheral Treg cells. Tamoxifen-mediated deletion of the *Cd28* gene reduces the number of Treg cells in the periphery [42]. This reduction is not due to differences in thymic export, but rather to impaired proliferation of Treg cells [42]. Likewise, Foxp3-cre-mediated deletion of CD28 in autoimmune disease models causes loss of suppressive activity by Treg cells [43]. CD28 signals induce expression of miR17-92 family members, leading to accumulation of antigen-specific Treg cells and maximal IL-10 production by Treg cells [44]. Treg-specific deletion of miR-17-92 causes exacerbated experimental autoimmune encephalomyelitis (EAE), an animal model of MS [44]. CD28 recruits Lck and activates NF-κB, leading to tTreg cell development [45]. In addition, CD28, together with the TCR, promotes expression of GITR, OX40, and tumor necrosis factor receptor 2 (TNFR2), leading to tTreg cell generation [46]. Costimulatory signals are also required to generate iTreg cells; the Lck-binding motif within the CD28 cytoplasmic domain is indispensable for this [47]. However, strong Lck signaling through CD28 inhibits iTreg cell differentiation, a role opposite to that played during tTreg cell development [48,49].

In addition to costimulatory molecules, T cells also express receptors that inhibit TCR signals; these are called co-inhibitory receptors. Co-inhibitory receptors attenuate and/or terminate activation signals initiated by stimulatory receptors. Treg cells express abundant co-inhibitory receptors such as CTLA-4, PD-1, and LAG-3 [50]. Since costimulatory and co-inhibitory pathways regulate T cell activation, they have been studied extensively in the context of autoimmunity [50]. In general, blocking co-inhibitory receptors increases immune responses, because it unrestrains T cell activity [50]; however, co-inhibitory receptors are shared by both conventional T cells and Treg cells. Although we still do not know how these pathways play Treg-specific roles, we do know that blocking these co-inhibitory pathways using anti-PD1 and anti-CTLA-4 antibodies promotes anti-cancer activity; such blocking antibodies are used widely for cancer treatment [51]. Thus, the same principles may apply with respect to regulation of immune cell activity and other areas of immune-related disease, such as chronic infection [52].

## 5. Cytokine Signaling

Cytokines are the most powerful determinant of CD4 T cell fate. As mentioned above, both Th17 and Treg cells require TGF-β signals. At the initial stage, TGF-β induces both Th17 and Treg cell programs [53]; however, the presence of IL-6 is a critical determinant of subsequent cell fate decisions. IL-6 drives Th17 cell differentiation by phosphorylating and activating STAT3, which then induces Th17-specific genes, such as *Rorc*, *Il17*, and *Il23r* [3,4,5,54,55]. STAT3 also inhibits Treg cell differentiation by downregulating TGF-β-induced expression of Foxp3 [5,56,57]. The effect of IL-6 is bolstered by other proinflammatory cytokines, including IL-1β, IL-21, IL-23, and TNF-α [3,53,58]. Although the combination of IL-6 plus TGF-β is a critical driver of Th17 cell differentiation, it is not sufficient for full acquisition of pathogenic properties by Th17 cells, since TGF-β plus IL-6 also induce IL-10 [59]. For pathogenicity, Th17 cells require an IL-23 signal along with IL-6 plus TGF-β, to induce IL-23 receptor expression [56,60,61].

On the other hand, TGF-β and IL-2 are essential for Treg cell differentiation. TGF-β signaling phosphorylates and activates the transcription factors Sma- and Mad-related protein (SMAD)2 and SMAD3 [62], which then bind to the *Foxp3* locus and induce expression of the *Foxp3* gene. IL-2 signaling is also important for Treg cell homeostasis [63,64]. IL-2 signaling phosphorylates STAT5, which binds to the *Foxp3* locus and induces expression of *Foxp3* [65]. However, TGF-β inhibits differentiation of Th1 and Th2 cells, and IL-2 inhibits that of Th17 cells [66,67].

## 6. Metabolic Pathways

Metabolic reprogramming and external signals that modulate metabolic pathways can affect the Th17/Treg balance. Naïve T cells need little energy and, therefore, utilize oxidative phosphorylation and fatty acid oxidation pathways [68]. In general, activated effector T cells become anabolic to meet the demands of cell proliferation and growth; therefore, they rely on glycolysis for ATP synthesis [69]. By contrast, Treg cells are catabolic; therefore, they metabolize fatty acids and amino acids, as well as glucose, and use oxidative phosphorylation to synthesize ATP [69].

The influence of metabolic reprogramming on T cell differentiation and function was discovered by examining mTOR. mTOR acts as an integrator of environmental signals supplied by growth factors, nutrients, oxygen, and energy levels [70]. When naïve T cells are activated, mTOR is activated and acts as a critical regulator that modulates T cell differentiation and function [71]. mTOR forms two multiprotein complexes: mTOR complex 1 (mTORC1) and mTOR complex 2 (mTORC2). Proper function of these complexes is required for upregulation of glycolysis and for differentiation into specific effector subsets. Deficiency of both mTORC1 and mTORC2 renders naïve CD4 T cells unable to upregulate the glycolytic machinery needed to support effector function, leading to a regulatory phenotype instead [68]. In addition, the mTOR inhibitor rapamycin promotes Foxp3 expression and expands existing tTreg cells [72,73]. Defective mTOR activity affects the Th17/Treg cell balance by increasing the sensitivity of T cells to TGF-β, thereby rendering the cells insensitive to the effects of proinflammatory cytokines on STAT3 signaling [74]. In ex vivo cultured cells and in human transplantation models, rapamycin shifts the Th17/Treg balance toward Tregs [75]. Although complete inhibition of mTOR leads to a shift toward Treg cells, inhibition of individual mTORC complexes produces different results. Mice deficient in mTORC1 activity cannot generate Th17 responses [76]. By contrast, inhibition of mTORC2 activity does not affect Th17 responses [76].

Th17 cells rely on acetyl-CoA carboxylase 1-mediated de novo fatty acid synthesis and the underlying glycolytic–lipogenic metabolic pathway for their differentiation [77]. By contrast, Treg cells depend on oxidative phosphorylation and importation of fatty acids from outside the cell [77]. Th17 cell differentiation can be enhanced by HIF1-α, which is a critical sensor of hypoxia and is responsible for mediating cellular responses to low oxygen. When HIF1-α is induced by mTORC1, it upregulates the glycolytic pathway and induces RORγt; however, it inhibits Foxp3, leading to Th17 cell differentiation [78].

## 7. Microbiota

The gastrointestinal tract has a very large surface area and makes contact with many foodborne and microbial antigens. The gut is colonized by a huge number of commensal microorganisms, collectively known as the microbiota. Since most antigens derived from food and from the commensal microbiota are not harmful, immune responses against such antigens must be suppressed. Thus, the gut is a place in which immune tolerance is paramount for maintenance of immune homeostasis. The gut is covered by dense layers of mucus. Although most microorganisms do not adhere to epithelial cells by passing through these layers, some pathogens or pathobionts attempt to do so.

Recent studies have begun to elucidate the way in which microorganisms influence the Th17/Treg balance [79]. Segmented filamentous bacteria (SFB) induce Th17 cell differentiation in the small intestine, and other tissues during autoimmune inflammation [80,81,82,83]. SFB antigens are presented to CD4 T cells by dendritic cells (DCs), leading to differentiation into SFB-specific Th17 cells [84,85]. SFB colonization boosts immune responses against the microbiota by inducing IgA, antimicrobial peptides, and proinflammatory cytokines [80,81,86]. Intestinal barrier immunity is regulated by the IL-23 receptor and IL-22 pathways. Breach of the intestinal barrier, or its penetration by microorganisms or their products, activates the IL-23 pathway and Th17 responses [87]. IL-23 also acts on type 3 innate lymphoid cells to stimulate epithelial cells to produce serum amyloid A proteins 1 and 2, leading to Th17 cell differentiation in the intestine [88].

*Bacteriodes fragilis* protects against experimental colitis by releasing polysaccharide A (PSA), which reduces production of IL-17 by immune cells in the intestine and promotes differentiation into IL-10-producing Foxp3^+^ Treg cells [89,90]. Likewise, *Bacteriodes thetaiotamicron* plays an anti-inflammatory role by controlling PPAR-γ trafficking between the nucleus and cytoplasm [91].

*Clostridia* induce Treg cells in the gut. Colonization of germ-free (GF) mice by a mixture of 46 *Clostridium* strains belonging to clusters XIVa and IV induces pTreg cells [92]. Moreover, a mixture of 17 strains belonging to *Clostidiales* clusters VI, XIVa, and XVIII induces Treg cells [93]. Furthermore, colonization of GF mice with a mixture of benign commensal microbiota triggers generation of Treg cells and inhibits Th17-mediated immune responses [94], suggesting that the microbiota play a vital role in Treg cell induction in the intestine.

Although it is not clear how commensal microorganisms induce Treg cells in the gut, the role of microbial metabolites in triggering Treg cell differentiation is becoming clearer. Short-chain fatty acids (SCFAs) produced by *Clostridales* induce Treg cell differentiation [95,96,97]. Oral administration of SCFAs contributes to Treg cell trafficking to the colon by inducing G-protein receptor (GPR) 15 [98]. SCFAs also induce the epigenetic status of Treg cells. Butyrate, a type of SCFA, inhibits proinflammatory cytokines by blocking histone deacetylases in macrophages and DCs [96,99], and by promoting acetylation of histone H3 in Treg cells at the *Foxp3* locus [97].

Disturbance of the normal microbiota is called dysbiosis. It is widely accepted that dysbiosis may be the cause of chronic inflammation associated with IBD [100], although the underlying mechanisms are not clear. Dysbiosis can affect the Th17/Treg balance in IBD. In the normal intestine, the microbial community is diverse and maintained by interactions with the host immune system; in particular, IgA and antimicrobial peptides that protect mucus layers outside of epithelial cells [101]. However, during dysbiosis this balance is disturbed, and overgrowth of pathobionts occurs. Pathobionts try to attach to or invade intestinal epithelial cells, thereby triggering innate signaling pathways such as the inflammasome, toll-like receptors, and nucleotide-binding oligomerization domain (NOD)-like receptors; this leads to production of proinflammatory cytokines (IL-1β, IL-6, IL-12, IL-18, and IL-23) [101], which then tip the Th17/Treg balance toward Th17 cells.

## 8. Plasticity of Th17 and Treg Cells

CD4 T cell subsets are not fixed; rather, they can change into other subsets when stimulated by different cytokines. This flexibility is called plasticity [102]. For example, when Th17 cells are stimulated by IFN-γ, they convert to Th1 cells. Although it is unclear whether Treg cells convert into effector subsets, many studies report that they can, particularly under lymphopenic or inflammatory conditions [103]. A study using lineage-tracing mice identified a substantial portion of exTreg cells (cells that once expressed Foxp3 but lost it later) in mice. When these exTreg cells were adoptively transferred, they induced diabetes and expressed proinflammatory cytokines (IFN-γ and IL-17), suggesting that they were activated memory cells [104]. Another study based on a similar approach shows that IL-17-expressing exTreg cells are responsible for inflammation associated with autoimmune arthritis [105]. By contrast, a study using different lineage-tracing mice showed an opposite result. In these mice, Foxp3+ cells hardly lost Foxp3 expression under both homeostatic and autoimmune inflammatory conditions, suggesting that Treg cells are highly stable [106]. Further studies are needed to resolve this issue.

On the other hand, transdifferentiation from Th17 cells to Tr1 cells has been reported. An experiment using lineage-tracing mice with Th17-mediated colitis revealed that Th17 cells acquire the signature transcriptional profile and potent suppressive functions of Tr1 cells [107].

Since RORγt and Foxp3 are lineage-determining transcription factors expressed by Th17 and Treg cells, respectively, transcriptional and post-transcriptional regulation of these proteins affects the Th17/Treg balance [108,109,110]. The stability of RORγt and Foxp3 is regulated by many post-transcriptional modifications, including ubiquitination, acetylation, and phosphorylation [110]. A recent study shows that reciprocal differentiation of Th17/Treg cells is also regulated by TEAD transcription factors TAZ and TEAD1 [111]. TAZ expressed by Th17 cells acts as a cofactor for RORγt, and destabilizes Foxp3, whereas TEAD1 expressed by Treg cells sequesters TAZ [111]. In addition to these proteins, others also affect the Th17/Treg balance. Transcription factor BACH2 suppresses effector T cell differentiation, while at the same time promoting Treg cell differentiation; indeed, BACH2-deficient mice show fulminant inflammation [112]. Transcription factor YY1 inhibits Treg cell function by preventing *Foxp3* transcription and transcriptional activity [113]. Transcription factor Batf3 shifts the balance toward Th17 by inhibiting transcription of *Foxp3* by repressing transcription of *Foxp3* [114]. Other factors are also thought to regulate the Th17/Treg balance; such factors may provide new therapeutic options for autoimmune diseases.

## 9. Autoimmune Diseases Caused by Dysregulation of the Th17/Treg Balance

Since differentiation of Th17 and Treg cells is regulated reciprocally by shared and different cytokines, and since each subset can convert to the other under certain inflammatory conditions, it is not surprising that the Th17/Treg balance plays a critically important role in many autoimmune diseases. Indeed, the Th17/Treg ratio is increased in patients with RA, psoriasis, MS, and IBD [115,116,117]. Among these, RA has been extensively studied from the perspective of the Th17/Treg balance. RA is characterized by chronic inflammation of the joint synovium, which results in destruction of bone and cartilage. Th17 cells are thought to be the main driver of pathogenesis by initiating inflammation via activation of many cell types within the inflamed joint, including macrophages, DCs, and synoviocytes. Accumulated evidence suggests that the Th17/Treg balance underlies the pathogenic mechanisms driving autoimmune diseases [118,119]. Therapeutic approaches designed to correct the balance have proven effective, and have been approved for the treatment of RA, psoriasis, psoriasis and psoriatic arthritis, ankylosing spondylitis, systemic lupus erythematosus, MS, and IBD [118,119]. Therapeutic approaches aimed at neutralizing Th17-related cytokines (including IL-6, TNF-α, IL-17, and IL-23) using monoclonal antibodies have been quite successful [118,119]. Monoclonal antibodies against the human IL-6R, tocilizumab and sarilumab, affect the ratio of Th17/Treg cells by reducing Th17 but increasing Treg cell levels in RA patients [120,121,122]. In addition, therapies designed to target RORγt, STAT3, Foxp3, and Foxo1 using small molecules are being developed [118,119]. Digoxin, an inhibitor of RORγt, ameliorates EAE and collagen-induced arthritis, an animal disease model of RA, by reducing the Th17/Treg ratio [123].

## 10. Conclusions and Perspective

**Figure 2:**
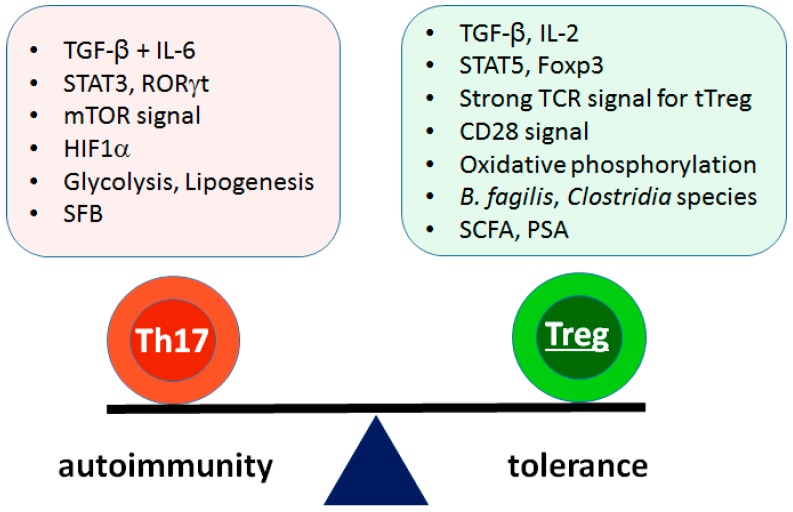
Contributing factors for reciprocal differentiation of Th17 versus pTreg cells. Differentiation of Th17 and pTreg cells are reciprocally regulated by many contributing factors. Cytokines are the most powerful determinants in the regulation. Other factors including TCR signal, costimulatory signal, metabolism, and microbiota also influence the balance. SFB: segmented filamentous bacteria, SCFA: short chain fatty acid, PSA: polysaccharide A.

Since many autoimmune diseases are driven by Th17 cells and suppressed by Treg cells, the balance between these cell types is critically important for pathogenesis, prognosis, and therapy. In addition to cytokine signals, this balance is affected by TCR and costimulatory signaling, metabolic pathways, and the microbiota (Figure 2). Treating autoimmune diseases by correcting this balance is effective, particularly for RA [119]. However, the drugs that work for RA are not equally effective against other autoimmune diseases [119], suggesting different etiologies and pathogeneses. Therefore, further studies should focus on identifying suitable therapeutic approaches for other autoimmune conditions. In addition, antigen-specific therapies are not widely available, and most therapeutic drugs have side effects. Approaches that target disease-specific antigens are needed.
